# The Effect of Compound *Sophora* on Fluorouracil and Oxaliplatin Resistance in Colorectal Cancer Cells

**DOI:** 10.1155/2019/7564232

**Published:** 2019-12-27

**Authors:** WeiHua Yin, GuPing Zhong, HuiZhen Fan, HongMei Xia

**Affiliations:** ^1^Departments of Oncology, The People's Hospital of Yichun Affiliated to Clinical Medicine School of Yichun University in Jiangxi Province, Yichun, Jiangxi 336000, China; ^2^Key Laboratory for Research on Active Ingredients in Natural Medicine of Jiangxi Province, Yichun University, Yichun 336000, China

## Abstract

Fluorouracil (5-FU) and oxaliplatin (L-OHP) are the most commonly used chemotherapy drugs for colorectal cancer, though resistance is common. Compound *Sophora* injection is a traditional Chinese medicine that can protect the liver against oxidation, improve immunity, and enhance sensitivity to chemotherapy; it may have an effect of reversing resistance in 5-FU- and L-OHP-resistant gastric cancer cells (5-FU/SW480 and L-OHP/SW480, respectively). A concentration gradient experiment was performed to identify a nontoxic dose of compound *Sophora* injection. 5-FU/SW480 and L-OHP/SW480 cells were treated with the nontoxic dose of compound radix Sophorae injection for 48 h, and changes in drug resistance to 5-FU and L-OHP were detected. Alterations in apoptosis and the cell cycle were assessed, as were the mRNA and protein levels of permeability glycoprotein (P-gp), annexin A1 (ANXA1), and ATP-binding cassette superfamily G member 2 (ABCG2). Flow cytometry showed a reduction in the number of cells in the G1 phase and an increase of cells in the S phase (*P* < 0.05). mRNA and protein expression of P-gp and ABCG2 was significantly higher in 5-FU/SW480 and L-OHP/SW480 cell lines, and ANXA1 expression decreased significantly (*P* < 0.05). Compound *Sophora* injection can reverse the drug resistance of 5-FU/SW480 and L-OHP/SW480 cell lines to 5-FU and L-OHP, respectively, possibly through a mechanism involving reduced expression of P-gp and ABCG2 but enhanced expression of ANXA1, which is the basis for the identification of clinical drug resistance in colorectal cancer.

## 1. Introduction

In recent years, the incidence of colorectal cancer has increased annually worldwide, becoming one of the most common malignant tumours. The incidence of colorectal cancer ranks third of all malignant tumours, and the fatality rate ranks fifth [1, 2]. Chemotherapy is a common treatment for colorectal cancer, though multidrug resistance (MDR) in tumors often leads to treatment failure. MDR (also known as multidrug hold) occurs when a tumour cell develops antitumour drug resistance, in which different chemical structures exert different actions. Different antitumour drugs also produce cross-resistance [3, 4]. Abnormal expression of drug-resistance proteins such as permeability glycoprotein (P-gp), annexin A1 (ANXA1), and ATP-binding cassette superfamily G member 2 (ABCG2) have been found in colorectal tumour tissues of patients with primary MDR, resulting in different levels of drug resistance to chemotherapy drugs in tumour cells [5–7]. Therefore, the search for effective drugs to reverse MDR has become a hot topic in the treatment of colorectal cancer, and an increasing number of researchers are paying attention to traditional Chinese medicine due to extensive advantages of low toxicity, high efficiency, and multitarget function [8]. Indeed, studies to date have shown the potential for the development of traditional Chinese medicine to treat tumour MDR. Compound radix Sophorae injection is a traditional Chinese medicine that exerts antioxidant effects, protects the liver, enhances immunity, and provides chemotherapeutic sensitivity [9–11]. The injection is prepared from extracts of radix *Sophora flavescens*, poriacocoscocos, and pachycocoscocos, and the main effective components are oxymatrine, sophorine, and matrine. Rasul et al. showed that compound matrine injection and its components, such as matrine and oxymatrine, had direct killing effects on various tumour cells, such as SGC-7901, HepG2, and BEL-7402, and could inhibit the invasion and metastasis of gastric cancer SGC-7901 cells [12–14]. Oxymatrine inhibits the proliferation of vascular endothelial cells [15]. In general, compound radix *Sophora flavescens* injection has been found to have a good antitumour effect in clinical applications. Combined chemotherapy can effectively reduce or stabilize the tumours, improve quality of life, and significantly alleviate pain due to cancer [16]. Compound *Sophora* injection has occasional adverse reactions to rashes in the clinic [16, 17]. However, it is not clear whether compound *Sophora* injection can reverse the resistance. In this study, the effects of compound *Sophora* injection on the expression of P-gp, ANXA1, ABCG2, and other drug-resistance proteins in drug-resistant colorectal cancer cell lines (fluorouracil (5-FU)/SW480 and oxaliplatin (L-OHP)/SW480) were studied [5–7], as were its effects on MDR, and the reversal mechanism was examined.

## 2. Materials and Methods

### 2.1. Cells

The colorectal cancer cell line SW480 was purchased from American Type Culture Collection (ATCC). 5-FU and L-OHP were both produced by Jiangsu Hengrui Pharmaceutical Co., Ltd. The compound radix Sophorae injection was obtained from Shanxi Zhendong Pharmaceutical Co., Ltd. The RPMI-1640 medium was purchased from GIBCO (Thermo Fisher Scientific, Inc., Waltham, MA, USA).

#### 2.1.1. Resistant Strains

Resistant strains were induced by a short-term method. At the logarithmic growth stage, most SW480 cells died after 1 h of incubation with 5-FU and L-OHP. The RPMI-1640 medium was added to the cells, which were washed twice with RPMI-1640 and further cultured. When the SW480 cells were again in the logarithmic growth phase, 5-FU or L-OHP was added for 1 h and the induction was repeated. After 8 months, the SW480 cell lines could be grown in the RPMI-1640 medium containing 5-FUor L-OHP, and a single-cell suspension of SW480 5-FU and L-OHP colorectal cancer cell substrains (5-FU/SW480 and L-OHP/SW480, respectively) was obtained. The resistance indexes of 5-FU and L-OHP were 24.26 and 25.31, respectively, as detected by the MTT assay.

#### 2.1.2. Reversal Experiments

At the logarithmic growth stage, 5-FU/SW480 and L-OHP/SW480 drug-resistant cells were inoculated into 96-well plates at a density of 1 × 10^5^ cells per well. After the cells had adhered to the plate, 20 *μ*L, 10 *μ*L, 5 *μ*L, 2.5 *μ*L, or 1.25 *μ*L of compound bitter *ginseng* injection was added to a final volume of 20 *μ*L. Next, 0 *μ*L, 10 *μ*L, 15 *μ*L, 17.5 *μ*L, and 18.75 *μ*L of PBS was added. The following control groups were established: no addition, blank control, solvent control, and drug (5-FU and L-OHP; positive control). The cells were cultured at 37°C in a 5% CO_2_ incubator for 48 h. To calculate the inhibitory rate, a concentration-inhibitory rate curve was generated by the MTT assay. The 50% inhibitory concentration (IC50) of compound radix Sophorae injection for 5-FU/SW480 and L-OHP/SW480 cells after 48 h of treatment was calculated as the nontoxic dose to reverse drug resistance. The IC50 values of 5-FU/SW480 and L-OHP/SW480 cells not treated with compound radix Sophorae injection were compared with those treated with the chemotherapy drugs. The drug resistance reversal multiple was calculated, where the reversal multiple is the value of IC50 without reversal divided by the value of IC50 after reversal.

#### 2.1.3. Apoptosis and the Cell Cycle

The drug-resistant strains in the logarithmic growth phase were inoculated into a 24-well cell culture plate. After the cells had adhered to the plate, RPMI-160 medium containing 5-FU and L-OHP was added. At the same time, the negative control group was prepared. After 48 h, 0.25% trypsin (without EDTA) was used to digest and collect the cells. Cells from each treatment group were collected by centrifugation, and annexin V and propidium iodide (PI) double staining was performed according to the manufacturer's instructions. Flow cytometry was also performed. A single-cell suspension was prepared with 70% ethanol, fixed for 2 h at 4°C, and incubated with 100 *μ*L of RNase A at 37°C for 30 min. Then, 400 *μ*L of PI stain was added and mixed, and the cells were placed in the dark at 4°C for 30 min. The cell cycle was examined by recording red fluorescence at an excitation wavelength of 488 nm.

#### 2.1.4. mRNA Expression

During logarithmic growth, total RNA from drug-resistant 5-FU/SW480 and L-OHP/SW480 cell lines was extracted with the TRIzol reagent. The extracted RNA (11 *μ*L) (less than 0.25 mg) was transferred to a new RNase-free EP tube, and 1 *μ*L of oligo-dT suspension and 1 *μ*L of dNTP mix (10 mM) were added and incubated at 70°C for 10 min (pyrolysis of the RNA secondary structure). The mixture was incubated on ice for 10 min (to let Oligotex combine with the mRNA), after which 4 *μ*L of 5x first-strand buffer, 1 *μ*L of 0.01 M DTT, 1 µL of RNase inhibitor, and 1 *μ*L of Superscript TMIII RT (invitrogen) were added and incubated at 42°C for 40 min. The mixture was then incubated at 70°C for 15 min, and the reaction was stopped at 94°C for 5 min. Using this cDNA as a template, P-gp, ANXA1, ABCG2, and *β*-actin were detected by PCR (primers were designed by ourselves and synthesized by Guangzhou Vingjun Company). The reaction conditions were as follows: 94°C for 2 min, 36 cycles of 94°C for 30 s, 58°C for 20 s, and 72°C for 1 min, followed by extension at 72°C for 5 min. The PCR products were examined on 1.0% agarose gel. The primer sequences used for each gene were as follows:

 
*β*-Actin F: 5′-GAAGTCGGAGTCAACGTATGA-3′, R: 5′-TTATGATGACTTCTTGTGCTA-3'
  P-gp F: 5′-ACGTAGTTGGTCCGTGAT-3′, R: 5′-GAGGTTCGCCAGTGGTAC-3'
  ANXA1 F: 5′-CCACAACTTCGCAGAGTG-3′, R: 5′-CAGAACGGAGACGCATAA-3'
  ABCG2 F:5′-CATGGTGTATAGACGCCTGAC-3′, R: 5′-GTCCATATGATGTTGATGACG-3'

#### 2.1.5. Drug-Resistance Proteins

On the basis of the above experiments, the cells were digested with trypsin and then fully lysed with prechilled lysis buffer. Next, 10% SDS-PAGE was performed, and electrophoresis was stopped when the dye front entered the optimal separation zone (approximately 2/3 of the gel). The proteins were transferred to a nitrocellulose (NC) membrane for 90 min, and the membrane was marked. The membrane was blocked in 5% skim milk and washed with TBST, and the appropriate primary antibody (against P-gp, 1 : 250; ANXA1, 1 : 1 000; or ABCG2, 1 : 400) was added and incubated at 4°C overnight with oscillation. After washing, the secondary antibody (1 : 500) (in 5% skim milk) was added and incubated for 2 h at room temperature on a shaking table. One drop of DAB concentrate per 2 mL of 0.02 M PBST was added, followed by 30% H_2_O_2_ (final concentration 0.03%) at a ratio of 1 : 1000. The reagents were mixed and added to the membrane, and the membrane was washed with distilled water after colour development to a satisfactory degree. Images were then obtained.

### 2.2. Statistical Methods

SPSS 13.0 software was used for statistical analysis. Each data point is expressed as the mean ± SD. A 2-sample *t*-test or a rank-sum test was used to compare the data, and Student's *t*-test or analysis or variance was applied to compare measurement data. *P* < 0.05 was considered statistically significant.

## 3. Results

### 3.1. Inhibitory Effect of Compound Radix Sophorae

**Figure 1:**
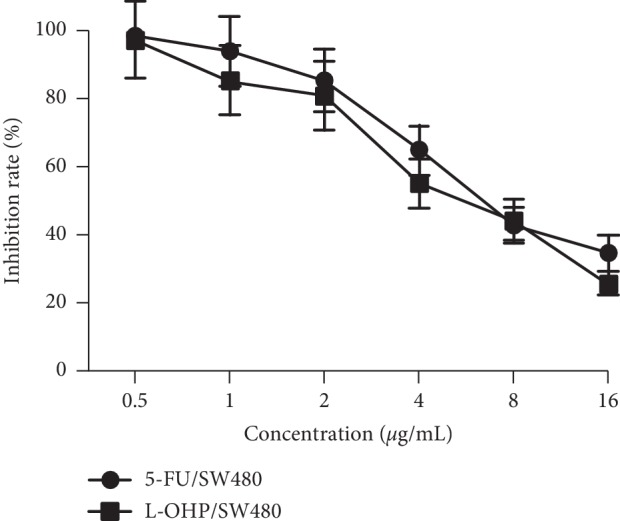
Cell proliferation assay. 5-FU/SW480 and L-OHP/SW480 drug-resistant cell strains were inoculated into 96-well plates at a density of 1 × 10^5^ cells per well, and 20 *μ*L, 10 *μ*L, 5 *μ*L, 2.5 *μ*L, and 1.25 *μ*L of compound matrine injection was added. After 48 h of culture, the cell inhibition rate gradually increased with an increase in the concentration of compound matrine injection. The 50% inhibitory rates of 5-FU/SW480 and L-OHP/SW480 cells were 0.84 and 0.89 g/mL, respectively, which were used as nontoxic doses to reverse drug resistance.

5-FU/SW480 and L-OHP/SW480 drug-resistant cell strains were inoculated into 96-well plates at a density of 1 × 10^5^ cells per well. After adherence, 20 *μ*L, 10 *μ*L, 5 *μ*L, 2.5 *μ*L, and 1.25 *μ*L of compound radix Sophorae injection was added.  Figure 1 shows that inhibition rates for the 5-FU/SW480 and L-OHP/SW480 cells rose with the increase in the concentration of compound radix Sophorae injection, and this inhibition correlated positively with the drug concentration. SPSS 13.0 software was used to calculate the IC50 values of 5-FU/SW480 and L-OHP/SW480 cells 48 h after compound radix Sophorae injection, which were 0.84 and 0.89 g/mL, respectively. These concentrations were used as nontoxic doses to reverse drug resistance.

### 3.2. Reversal Ability of Compound Radix Sophorae

The IC50 value of 5-FU to 5-FU/SW480 cells was 5.41 ± 0.12 g/mL, and the IC50 value of L-OHP to L-OHP/SW480 cells was 6.15 ± 0.24 g/mL. The IC50 values of 5-FU and L-OHP for the corresponding drug-resistant cells were 2.62 ± 0.18 g/mL and 3.13 ± 0.14 g/mL, respectively, after the addition of nontoxic doses of compound radix *Sophora flavescens* injection (0.84 and 0.89 g/mL, respectively). The IC50 values decreased (*P* < 0.05), and the drug resistance reversion multiples were 2.07 and 1.97, respectively. This result indicated that compound radix Sophorae root injection had a drug resistance reversal effect on 5-FU/SW480 and L-OHP/SW480 cells.

### 3.3. Analysis of Apoptosis and the Cell Cycle

**Figure 2:**
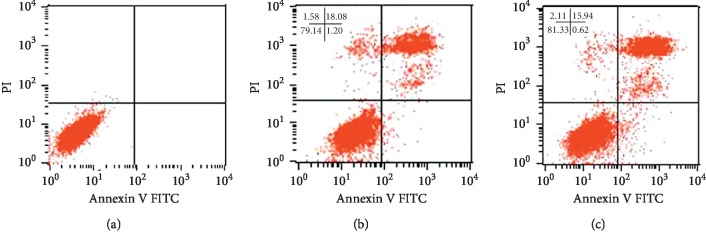
Characterization of apoptosis and the cell cycle. The drug-resistant cell strains were cultured in RPMI-160 medium (containing 5-FU and L-OHP) and plated in 24-well culture plates according to the above descriptions and incubated with a nontoxic dose of compound radix Sophorae injection for 48 h. Flow cytometry detection showed that the number of 5-FU/SW480 and L-OHP/SW480 drug-resistant cells in the G1 phase was decreased compared with that of the control group, and the numbers of cells in the S phase and G2 phase were significantly increased (*P* < 0.05; Table 1). Apoptosis increased slightly, though the difference was not significant (*P* > 0.05; Figure 2). (a) None, (b) 5-FU/SW480, and (c) L-OHP/SW480.

Cell cycle analysis was performed on 5-FU/SW480 and L-OHP/SW480 cells, which served as control groups. The numbers of 5-FU/SW480 and L-OHP/SW480 cells treated with compound bitter *ginseng* injection in the G1 phase were decreased compared with the control group, whereas the number of cells in S and G2 phases were significantly increased (*P* < 0.05; Table 1). Apoptosis increased slightly in 5-FU/SW480 and L-OHP/SW480 cells, though the difference was not significant (*P* > 0.05; Figure 2).

### 3.4. mRNA Detection in Drug-Resistant Strains

**Figure 3:**
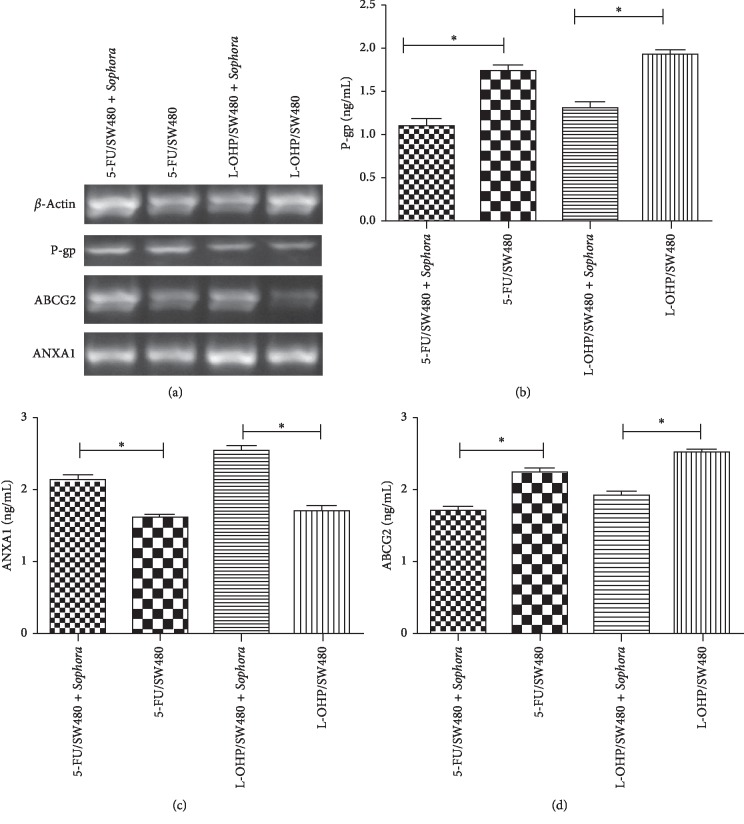
Effect of compound radix Sophorae injection on the mRNA levels of genes. 5-FU/SW480 and L-OHP/SW480 cells were incubated with a nontoxic dose of compound radix Sophorae Flavescentis injection for 48 h as described above. P-gp and ABCG2 mRNA expression levels were significantly decreased in the two drug-resistant strains (5-FU/SW480 and L-OHP/SW480). ANXA1 expression was significantly increased, as determined by RT-PCR (*P* < 0.05).

The mRNA expression levels of P-gp and ABCG2 in 5-FU/SW480 and L-OHP/SW480 cells were significantly decreased after the addition of a nontoxic dose of compound radix Sophorae injection. However, ANXA1 expression increased significantly (*P* < 0.05), as shown in Figure 3.

### 3.5. Drug-Resistance Protein Expression Detection

**Figure 4:**
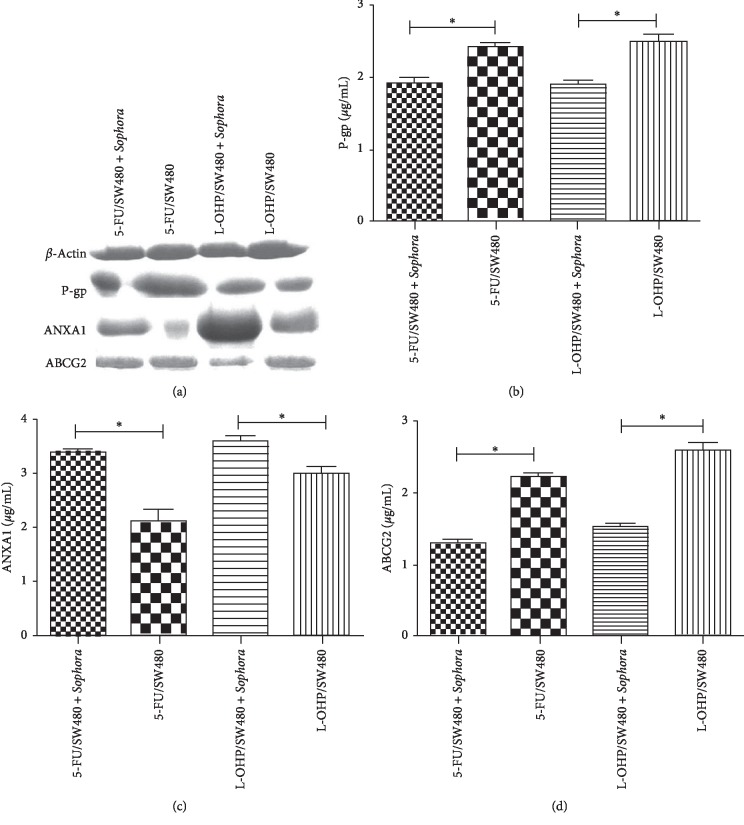
Effect of compound radix Sophorae injection on protein levels. The drug-resistant 5-FU/SW480 and L-OHP/SW480 cell lines in logarithmic growth phase were cultured with a nontoxic dose of compound radix Sophorae for 48 h The mRNA expression levels of P-gp and ABCG2 in the two drug-resistant strains (5-FU/SW480 and L-OHP/SW480) were significantly decreased, as determined by western blotting. ANXA1 expression was significantly increased after treatment with the compound radix Sophorae (*P* < 0.05).

Western blot analysis was used to detect the expression levels of related proteins. Without the addition of a nontoxic dose of compound bitter *ginseng* injection, the expression levels of P-gp and ABCG2 in the 5-FU/SW480 and L-OHP/SW480 cell lines were increased. But the expression levels of ANXA1 decreased significantly (*P* < 0.05; Figure 4).

## 4. Discussion

Chemotherapy is an important means of treating colorectal cancer, but many patients show MDR during the course of chemotherapy, which is the main reason for treatment failure [3, 4]. Chemotherapy for colorectal cancer consists mostly of three drugs, 5-FU, oxaliplatin. and irinotecan, which are combined in clinical practice [2], but treatment is difficult if resistance occurs [18]. Traditional Chinese medicine is expected to be an ideal agent for reversing MDR after the failure of chemotherapy for tumours due to its low toxicity, good stability, and unique effects [8, 9, 19]. In fact, identification of a Chinese medicine preparation that is sensitive, reduces toxicity, shows synergistic and complementary effects with drugs, and reduces drug resistance is urgently needed. Compound Sophorae Flavescentis injection is a traditional Chinese medicine that has antioxidant and antitumour properties and improves immunity [10, 20]. In the treatment of colorectal cancer, compound radix Sophorae injection, which can reduce the toxicity and side effects of chemotherapy drugs, improves the short-term efficacy of chemotherapy and the quality of life of patients [8]. Our preliminary research reveals that it can also reduce the toxicity and side effects of drugs and radiotherapy for colorectal cancer and has certain sensitizing functions for radiotherapy and chemotherapy in the treatment of colorectal cancer.

Chemotherapy resistance is a very common phenomenon in patients with colorectal cancer. It is believed that the mechanism of MDR involves two aspects:(1) membrane glycoproteins, energy-dependent drug efflux pumps that can reduce the concentrations of drugs in the cell or form a compartmentalized distribution, resulting in drug resistance, including P-gp, ABCG2, and MRP [5, 21], and (2) enzyme-mediated resistance (e.g., topoisomerase (toPo), glutathione transferase (GST), and protein kinase C (PKc)or abnormal expression of apoptotic regulatory genes, though antitumour drugs mainly play a role by inducing apoptosis). Among these factors, the Bcl-2 family, p53, and c-myc are involved in resistance [22, 23]. Compound radix Sophorae injection has antioxidant ability, enhances the function of GST and regulates the caspase pathway to induce apoptosis. Therefore, compound radix Sophorae root injection has a certain effect of reversing MDR in chemotherapy, and its mechanism is related to inhibition of the expression of the drug-resistance protein P-gp. This study showed that the IC50 values of 5-FUin 5-FU/SW480 cells and L-OHP in L-OHP/SW480 cells were 5.41 ± 0.12 g/mL and 6.15 ± 0.24 g/mL, respectively, with drug resistance reversion multiples 2.07 and 1.97, respectively. Thus, compound radix Sophorae root injection had a certain drug resistance reversal effect on 5-FU/SW480 and L-OHP/SW480 cells. Compound radix Sophorae root injection induces tumour cell apoptosis, which is one of the mechanisms for reversing drug resistance. ANXA1 is the first member of the family of membrane-linked proteins that inhibits the transcription of nuclear factor kappa B (NF-*κ*B) by blocking its binding to DNA, thereby inhibiting the proliferation of tumour cells and promoting their apoptosis [24]. Therefore, it is believed that the downregulated expression level of ANXA1 in drug-resistant cell lines leads to a decrease in tumour cell apoptosis, which further reduces the sensitivity of tumour cells to drugs and leads to resistance [6]. Our apoptosis (cell cycle) experiments showed that a nontoxic dose of compound radix Sophorae injection reduced the number of cells in the G1 phase, significantly increased the number of cells in the S phase, significantly increased ANXA1 expression, and slightly increased the rate of apoptosis following treatment, though the difference was not significant (*P* < 0.05), as shown in Figure 2.

Resistance genes and proteins are an important reason for the occurrence of drug resistance in tumour cells, and P-gp and ABCG2 are the most common proteins in this regard. The dependence of ATP is due to cell membrane transporters, which are energy-dependent drug efflux pumps that can actively pump drugs out of tumour cells. This process reduces the drug concentration or compartment distribution and results in a decline in the effective concentration of the drug in the cells, prompting them to develop properties of resistance [25]. The ABC transporter can transport drugs from the cytoplasm to the extracellular environment using the energy from ATP hydrolysis [26]. Studies have confirmed that ABCG2 is closely related to the drug resistance of breast cancer and nonsmall cell lung cancer [27–29]. At present, researchers believe that P-gp and ABCG2 can pump most chemotherapy drugs out of the cell, resulting in a reduction in the intracellular drug concentration and showing that tumour cells possess extensive drug resistance mechanisms, namely, MDR [30]. The results of this study showed that the 5-FU/SW480 and L-OHP/SW480 drug-resistant cell lines mainly developed chemical drug resistance by upregulating expression of P-gp, ABCG2, and other drug-resistance proteins. However, compound radix Sophorae injection reversed drug resistance in these cells by downregulating expression of P-gp, ABCG2, and other drug-resistance proteins.

In summary, compound radix Sophorae injection can reverse the resistance of 5-FU/SW480 and L-OHP/SW480 drug-resistant cells to 5-FU and L-OHP, respectively, and the reversal mechanism may be related to the expression of drug-resistance proteins, such as P-gp. Therefore, compound radix Sophorae injection can reduce the occurrence of drug resistance in colorectal cancer cells, increase the sensitivity of tumours to chemotherapy drugs, and effectively increase the short-term clinical effective rate among patients with drug-resistant tumours, which is worthy of clinical promotion.

## Figures and Tables

**Table 1:** Effect of compound radix Sophorae injection on the cell cycle.

Groups	G1	S	G2
5-Fu/SW480 + *Sophora*	37.43 ± 2.25^*∗*^	47.62 ± 3.47^*∗*^	15.72 ± 0.96^*∗*^
5-Fu/SW480	45.36 ± 2.49	41.42 ± 2.58	15.13 ± 1.18
L-OHP/SW480 + *Sophora*	36.61 ± 1.83^*∗∗*^	46.92 ± 2.13^*∗∗*^	17.69 ± 1.26^*∗∗*^
L-OHP/SW480	44.56 ± 3.29	40.69 ± 2.43	14.86 ± 0.92

^*∗*^ *P* < 0.05; ^*∗∗*^*P* < 0.05.

## Data Availability

All data supporting the findings of this study are included in this article.
